# The Effect of Hookah Use on Buccal Mucosa: Evaluation of Repair Index

**DOI:** 10.31557/APJCP.2019.20.4.1109

**Published:** 2019

**Authors:** Mehrdad Taghibakhsh, Sareh Farhadi, Afsaneh Babaee, Maryam Sheikhi

**Affiliations:** 1 *Department of Oral Medicine,*; 2 *Department of Oral and Maxillofacial Pathology, Faculty of Dentistry, Tehran Medical Sciences, Islamic Azad University,*; 3 *Dentist, Tehran, Iran.*

**Keywords:** Micronucleus, Repair Index, buccal mucosa, Hookah

## Abstract

**Background and aim:**

Cigarettes, hookah, and tobacco are the most important etiologic factors for oral cancers and dysplastic lesions. This study was undertaken to determine the correlation between hookah use and the percentage of cells with micronucleus, karyorrhexis, karyolysis, and broken egg in the buccal mucosa; and secondly to compare hookah user and non-user in terms of repair index.

**Materials and methods:**

The present historical cohort study was carried out on 72 samples taken from 36 hookah users and 36 control subjects. Smear samples were obtained from participants’ buccal mucosa for cytological evaluation using Papanicolaou technique. Then, the percentages of cells with micronucleus, karyorrhexis, karyolysis, and broken egg were recorded and the repair index was calculated. Data were analyzed using Mann-Whitney U test.

**Results:**

A total of 72 samples taken from 36 hookah users and 36 control subjects were evaluated. The means of micronucleus scores in the buccal mucosa cells of hookah users and controls were 10.7±2.6 and 5.8±2.0, the karyorrhexis scores in the hookah users and controls were 0.1±0.06 and 0.04±0.06, and the karyolysis scores in hookah users and controls were 0.16±0.05 and 0.08±0.06, respectively. These differences were statistically significant between hookah users and controls (P<0.001). The broken egg score was 0.66±0.07 for the hookah users and 0.03±0.04 for the control group, revealing a statistically significant difference (P<0.036). Finally, the repair index values were 0.03±0.01 and 0.05±0.13 in hookah users and controls, respectively. This difference was also significant (P<0.026).

**Conclusion:**

The percentages of cells with micronucleus, karyorrhexis, karyolysis, and broken egg in the buccal mucosa of hookah users were significantly higher than those in control group; in addition, the repair index of the buccal mucosa cells in hookah users was significantly lower than that in the control group.

## Introduction

Cancers have been known as life-threatening conditions all over the world. The diagnosis of oral cancers is still difficult and almost half of the individuals diagnosed with oral cancers die. Undoubtedly, the first step in the treatment is early diagnosis, especially in high-risk subjects (Cançado et al., 2004; Sargolzaei et al., 2014). Tobacco smoke and use of hookah are the most important etiologic factors for oral cancers and risk factors for dysplastic lesions. Some studies have reported that the tobacco smoke from hookah contains toxic agents such as carbon monoxide, heavy metals, and carcinogenic chemical agents (Siefi et al, 2014). Hookah smoke contains polycyclic aromatic hydrocarbons at a 20-fold concentration and heavy polycyclic aromatic hydrocarbons at a 50-fold concentration compared to cigarette smoke. In addition, the amount of carbon monoxide produced by hookah has been reported to be 5 times higher than that of cigarette. In addition, a study showed that during 45 minutes of hookah use, smoke is produced 40 times higher than that with cigarette smoking, which increases several folds the potential to induce disease (El-setouhy and Loffred, 2008).

In addition, lung cancers and periodontal and respiratory diseases have been reported at a higher rate in hookah users compared to normal individuals. Many studies have confirmed the relationship between cigarette smoke and oral cancers. Several review studies and reports have confirmed an increase in the risk of oral cancers due to the use of hookah. However, no strong relationship was reported between these two factors (Rabiei et al., 2014; Shafagoj et al., 2002).

On the other hand, some believe that filtration of hookah smoke through water decreases its nicotine. However, contrary to this belief, studies have shown that only 5% of nicotine is removed by water. Furthermore, hookah users might have a tendency to increase the duration of smoking, resulting in an increase in the concentration of nicotine in their bloodstream. Therefore, considering the aforementioned deleterious effects of hookah use and the results of a recent study, it seems that the use of this smoking device might induce changes in the oral mucosa. However, conducting further studies with larger samples size and evaluating other confounding factors are recommended (Rastam et al., 2004; Mary et al., 2011).

Evaluation of nuclear changes in the oral mucosa cells of individuals who use tobacco is one of the most non-invasive and fastest methods for the diagnosis of oral malignancies (El-setouhy et al., 2008). Injuries to the genome are possibly the main etiologic factors for induction of developmental and degenerative conditions and development of cancer. Nuclear changes occur in the early stages of cancer. Nuclear changes in the buccal mucosa cells were first reported in this field by Stich and Rosin (1983) and currently they are used as a biomarker for genetic injuries in many cases (Palaskar et al., 2010; Farhadi et al., 2016; Farhadi et al., 2017), which makes it possible to evaluate nuclear changes in cells encountering carcinogens before the emergence of clinical symptoms of cancer (Stich et al., 1984). Nuclear changes are evaluated in cytology samples from the buccal mucosa of patients; this is a simple, non-invasive, and rather painless technique (Saeed et al., 2012). 

Some studies have evaluated nuclear changes, including micronucleus, in smokers (Kashyap and Reddy, 2012). There is limited number of studies on hookah users in this respect. Therefore, the present study was undertaken to evaluate the relationship between hookah use and the percentage of cells with micronucleus, karyorrhexis, karyolysis, and broken egg in the buccal mucosa; and secondly to compare hookah user and non-user in terms of repair index.

## Materials and Methods

In the present historical cohort study, the participants were selected using objective-based sampling technique. The statistical population consisted of individuals presented in hookah cafes in Tehran in 2017. The inclusion criteria were no history of smoking, alcohol and drug consumption, systemic disease, head and neck radiotherapy, and exposure to chemical agents. Data were collected using interviews and completing a datasheet and by collecting samples from the buccal mucosa of the participants. Samples were taken using a wet tongue depressor and evaluated under a light microscope after staining.

The following nuclear changes were evaluated in the present study:

**Micronucleus (MN):** The criteria used to evaluate MN were introduced by Schmidt. According to Schmidt, MN is similar to the cell nucleus but it has a small size. MNs are round to oval in shape and have a clearly visible periphery and the same color as that of the main nucleus of the cell. However, their dimension is one-third of that of the main nucleus (Kumboj and Mahajan, 2007).

**Karyorrhexis (KR):** KR refers to a form of nuclear changes in which the nucleus becomes pyknotic or rather pyknotic and segmented and the nucleus of the necrotic cell completely disappears over time (1 or 2 days) (Kumar et al., 2010).

**Karyolysis (KL):** In KL, the basophilic appearance of the chromatin disappears. These changes possibly indicate the DNAse activity (Jalayer Naderi et al., 2017). 

**Broken egg (BE):** BE refers to changes in the nucleus resulted in a phenomenon, described as ‘nuclei that appear cinched’ (Tolbert et al., 1992). 

To determine the counts of nuclear changes, the criteria introduced by Tolbert et al., (1992) were used.

In order to carry out cellular evaluations, some fields were selected randomly and 500 cells were counted at ×400 magnification under a light microscope (Nikon) by an oral pathologist and the results were reported as percentages. Then, for more precise evaluation of nuclear changes the repair index (Farhadi et al., 2017) was calculated for each sample using the following formula and the data recorded in the datasheet:

Repair index = (KR+KL)/(MN+BE) 

This index may exhibite the ratio of nuclear changes presented more in damage (MN and BE) and those that are evident both in apoptosis procedure and carcinogenic damages (KR and KL) (Farhadi et al., 2017) .

Data were analyzed using Mann-Whitney U test.

**Figure 1:**
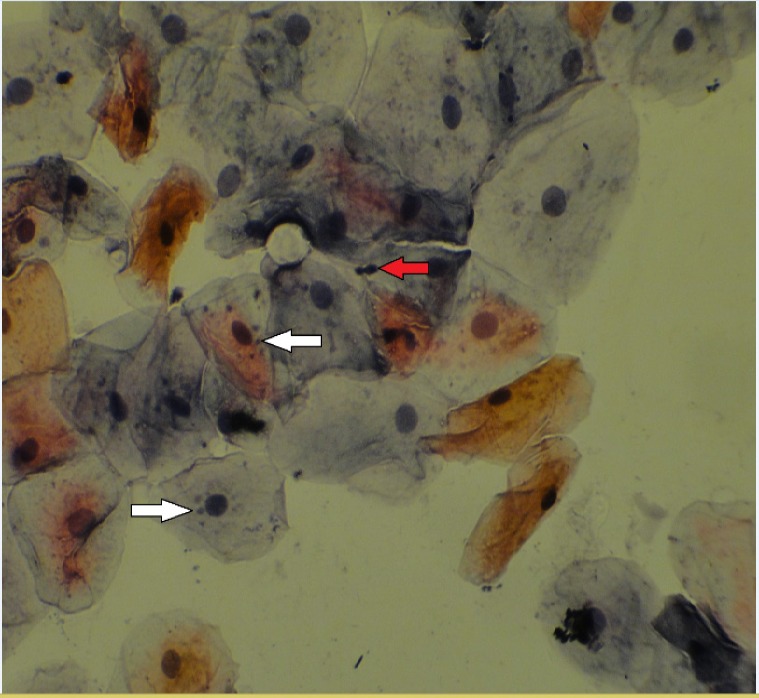
Cytological Examination Show Micronucleus (White Arrows) and Broken Egg (Red Arrow) by x400 Magnification

**Figure 2:**
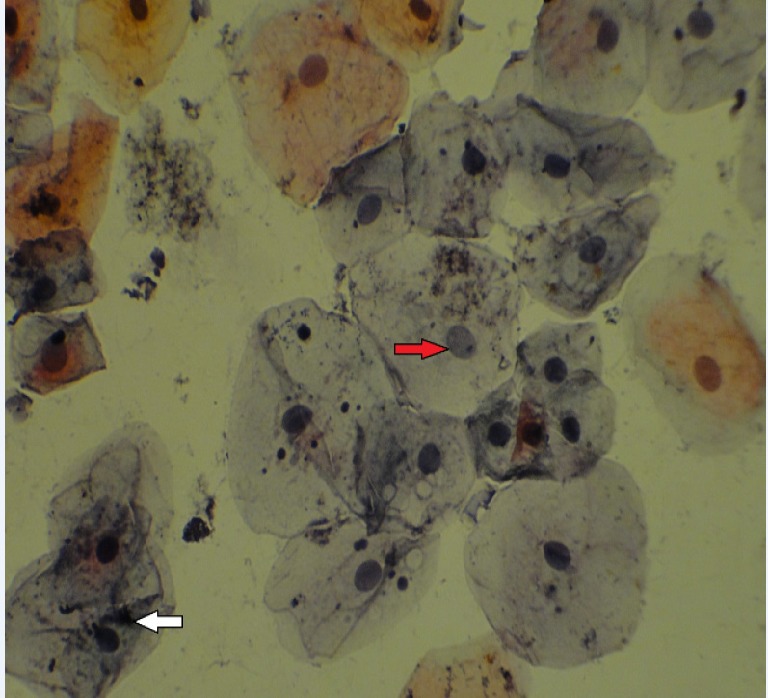
Cytological Examination Show Karyorrhexis (White Arrow) and Karyolysis (Red Arrow) by x400 Magnification

Figures 1 and 2 show nuclear changes at ×400 magnification.

## Results

The present study was carried out on 72 participants, consisting of 36 hookah users and 36 subjects who did not use hookah as a control group. All the participants were male and it was supposed that they had similar socioeconomic status because they had referred to the same place. The participants’ means of age were 27.3±5.9 years in the hookah group and 29.9±6.1 years and in the control group, indicating no significant difference between two groups.

**Table 1:** Comparison of Nuclear Changes Across Two Groups

Participants	MN (%)	KR (%)	KL (%)	BE (%)
Hookah users (n=36)	5.8±2.0	0.04±0.06	0.08±0.06	0.03±0.04
Control group (n=36)	10.7±2.6	0.1±0.06	0.16±0.05	0.06±0.07
Test result	P<0.001	P<0.001	P<0.001	P<0.036

**Table 2:** Comparison of Repair Index (RI) in Two Groups

Participants	RI value
Hookah users (n=36)	0.05±0.13
Controls (n=36)	0.03±0.01
Test result	P<0.026

As shown in Table 1, the mean of MN percentage was 1.8 folds higher in the hookah group (10.7) compared to the control group (5.8) (P<0.001). The mean percentages of KR in the control and case groups were 0.04 and 0.1, respectively, which was 2.5 folds higher in the case group compared to the control group (P<0.001). In addition, the mean percentages of KL in the control and case groups were 0.08 and 0.16, respectively, which was 2 folds higher in the control group compared to the case group (P<0.001). Finally, the mean percentages of BE in the control and case groups were 0.03 and 0.06, respectively, which was 2 folds higher in the case group compared to the control group (P<0.036). The repair index in the control and test groups were 0.05 and 0.03, respectively (Table 2), which was 40% higher in the control group compared to the case group (P<0.026).

## Discussion

The results of the present study showed that the mean percentages of buccal mucosa cells with MN, KL, KR and BE were higher in hookah users compared to control group. On the other hand, the repair index of the buccal mucosa cells in hookah users was significantly lower than that in the control group. 

Consistent with the results of the present study, a study by El-setouhy and Loffred, (2008) showed significant differences between hookah users and a control group with respect to the percentages of cells with MN; however, they did not consider any inclusion criteria for assigning to the control and case groups. Similarly, in studies by Seifi et al., (2014) and Jalayer-Naderi and PourPasha, (2017) on 3 groups consisting of hookah users, cigarette smokers, and control subjects, more nuclear changes were observed in the smokers and hookah users compared to the control subjects; however, these nuclear changes were more prominent among cigarette smokers (Jalayer-Naderi et al., 2017). In the studies above, the study groups had not been matched in relation to the variables.

In aforementioned studies, the size of the nucleus and its ratio to the cytoplasm, percentages of KR and KL and pyknosis and inflammation and candidiasis were evaluated. However, in the present study, a new index known as repair index was calculated in order to summarize the results of nuclear changes. Therefore, the present study used an innovative technique to report results. Although comparison of the details of the results in relation to KR in the present study and the study by Jalayer-Naderi and PourPasha, (2017) appears to be consistent in relation to KR, Jalayer-Naderi and PourPasha, (2017) did not report any significant differences between the 3 studies groups, which is different from the results of the present study. Such a discrepancy might be attributed to the use of different staining techniques; Jalayer-Naderi and PourPasha, (2017) used Feulgen staining technique and we used Papanicolaou staining technique.

Other studies have been carried out on the effects of tobacco. For instance, Jalayer-Naderi et al., (2012) reported significant differences in the means of cells with MN percentage in cigarette smokers and non-smoking group. Consistent with the results of the present study, Farhadi et al., (2017) and Sharma et al., (2013) indicated the deleterious effects of tobacco use on the oral mucosa. Given few studies on the use of hookah, the results of the present study might be a turning point for achieving more comprehensive and definitive findings in this regard.

Hookah smoke contains 20 folds of polycyclic aromatic hydrocarbons and 50 folds of heavy polycyclic aromatic hydrocarbons compared to cigarette smoke. In addition, a hookah has been reported to produce carbon monoxide 5 folds higher than that by cigarette. In addition, a study showed that during 45 minutes of hookah use, the volume of smoke produced was 40 times that of cigarette, increasing the potential to induce diseases (Rastam et al., 2004).

Moreover, lung cancer as well as respiratory and periodontal diseases incidences were reported at a higher rate in hookah users compared to normal subjects. The relationship between cigarette smoke and oral cancers has been confirmed by many studies. Several review studies and reports are available, indicating an increase in the risk of oral cancers due to the use of hookah (El-setouhy and Loffred, 2008). Although no strong relationship was reported between these two factors in some studies, others reported decreased nicotine content after filtration of smoke by water. However, some others believe that only 5% of nicotine is removed by water. In addition, hookah users might be interested in increasing the duration of smoking, which results in an increase in the concentration of nicotine in their bloodstream. Therefore, considering the adverse effects of hookah use discussed above and the results of the present study, it can be concluded that the use of hookah may give rise to changes in the oral cavity mucosa. However, further studies are recommended using larger sample sizes in order to evaluate other confounding variables (Martinasek et al., 2011). Furthermore, given the importance of nuclear changes especially MN as an biomarker (Kumboj and Mahajan, 2007) and considering the results of this study, the evaluation of nuclear changes in exfoliated buccal mucosa cells of hookah users can be useful for screening of oral dysplastic and malignant lesion, although more studies are mandatory in this issue. 

In conclusion, in this study, the means of percentage cells with MN, KL, KR, and BE were higher in the buccal mucosa of hookah users compared to the control group; whereas, the repair index of the buccal mucosa cells in hookah users was significantly lower than that in the control group.
